# Perceived faith-oriented public-space environments, activity participation, and social connectedness among older adults in Quanzhou Ancient City

**DOI:** 10.3389/fpubh.2026.1892546

**Published:** 2026-09-23

**Authors:** Yi Han, Bo Lin, Huan Ding, Bowen Deng

**Affiliations:** 1 School of Design & Innovation, Xiamen University Tan Kah Kee College, Zhangzhou, China; 2 Institute of Education, Xiamen University, Xiamen, China; 3 Art Center, Chiway Education Group, Shanghai, China

**Keywords:** activity participation, faith-oriented public space, historic district renewal, older adults, social connectedness

## Abstract

Older adults’ perceptions of urban public spaces are associated with their outdoor activities and social interactions, whereas locally embedded folk culture is associated with distinctive patterns of spatial engagement. In the context of historic city renewal, understanding how culturally embedded environments are associated with social connectedness among older adults is essential. This study examines how environmental perceptions of public spaces are associated with social connectedness among older adults and tests the indirect associations through activity participation. Using Licheng District of Quanzhou Ancient City as the study area, a structural equation model was developed to analyze the path relationships among five dimensions of environmental perception—activity-oriented, landscape-oriented, accessibility-oriented, communicative, and faith-oriented elements—activity participation, and social connectedness. Data were obtained from two repeated cross-sectional surveys conducted in October 2020 and January 2024 (*n* = 620) and analyzed using structural equation modeling. After adjustment for gender, age, educational attainment, household composition, and religious affiliation, all five dimensions of environmental perception were positively associated with stronger social connectedness: activity-oriented (*β* = 0.100, *p* = 0.017), landscape-oriented (*β* = 0.157, *p* < 0.001), accessibility-oriented (*β* = 0.169, *p* < 0.001), communicative (*β* = 0.125, *p* = 0.001), and faith-oriented perception (*β* = 0.091, *p* = 0.022). Faith-based folk activities (*β* = 0.169), non-faith folk activities (*β* = 0.145), and non-folk activities (*β* = 0.180) were also positively associated with social connectedness (all *p* < 0.001). Bootstrap analyses identified activity-specific indirect associations between environmental perception and social connectedness. Given the repeated cross-sectional design, the findings indicate statistical associations rather than causal effects.

## 1 Introduction

With the rapid growth of ageing populations worldwide, social isolation has become an important public health concern because it is associated with adverse physical and mental health outcomes among older adults (1). In China, nationally representative data from the China Health and Retirement Longitudinal Study indicated that the standardized prevalence of social isolation among middle-aged and older adults was 38.09% in 2011, 33.66% in 2013, 39.13% in 2015, and 39.95% in 2018 (2). Social disconnectedness and perceived isolation represent related but distinct dimensions of social isolation, and both have been independently associated with poorer self-rated physical health (3). In contrast, stronger social connectedness, reflected in supportive interpersonal relationships, social participation, and a sense of belonging, has been associated with lower loneliness, greater engagement in life, and better health and well-being in later life (3, 4). Urban public spaces constitute important settings for older adults’ daily activities and social encounters and may provide opportunities to maintain interpersonal ties and social participation (5, 6). Examining how older adults perceive these everyday environments is therefore important for understanding the environmental conditions associated with social connectedness in later life.

Public spaces are important settings for a wide range of social activities and have been associated with stronger social connectedness among older adults, including greater opportunities for outdoor activity, lower loneliness and emotional distress, broader social networks, and a stronger sense of belonging (7). Based on a study of residents in new housing developments in metropolitan Perth, Francis et al. (7) examined how different types of public spaces, including public open spaces, community centers, schools, and shops, were associated with sense of community. Their findings indicated that public-space characteristics were associated with social well-being through physical provision, perceived safety, participation, social interaction, and neighborhood attachment. Previous studies have examined this issue from the perspectives of individual characteristics, the physical environment of public spaces, and the perceptual mechanisms associated with these environments. These studies indicate associations between public- and green-space environments and older adults’ social well-being, including relationships involving activity participation and social engagement (8). Existing research has shown that social connectedness is associated with a range of individual factors, including age, sex, educational attainment, household composition, and self-rated health (9). The physical environment of public spaces is commonly measured using quantifiable indicators such as the proportion of public space within a given area, the diversity of activities that a space can accommodate, and the level of accessibility and convenience (10). A larger proportion of public space, greater capacity to accommodate diverse activities, and closer proximity to older adults’ residences have been associated with higher levels of social connectedness (11, 12).

Environmental perception refers to individuals’ subjective feelings and psychological evaluations of their surrounding environment and environmental change, and it provides an important psychological basis for environmental behavior, activity participation, and social participation (13). In recent years, increasing attention has been paid to older adults’ perceptions of physical environmental attributes, and studies have shown that factors such as a suitable microclimate, green coverage, and facility diversity have been associated with the formation and maintenance of social networks (14). Researchers have also examined the relationship between subjective perceptions of public spaces and individual well-being, showing that perceived safety and spatial diversity are significantly associated with older adults’ mental health and social engagement (15). Further quantitative studies have found that preferences for spatial diversity and a favorable social activity atmosphere are associated with more frequent social interaction and stronger social connectedness in public spaces (16, 17).

Folk beliefs and related activities, which are often embedded in local public spaces, may provide culturally embedded opportunities for participation and social interaction among older adults (18). Folk activities typically have collective and social characteristics and have been associated with emotional exchange and social interaction among older adults (19). Their cyclical recurrence is likewise associated with sustained interaction and social cohesion among community members (20). Alongside kinship-based interaction, geographically rooted interaction is also prominent in these activities, indicating associations with local networks and regional belonging (21).

Taking the “pujing” system of Quanzhou Ancient City as an example, a jing represents not only a localized belief system but also a socio-spatial network constructed through temple-based rituals and territorial processions. Within this network, religious activities are associated with local community cohesion and older adults’ social participation (22). This bottom-up traditional community structure is characterized by a belief-based social network centered on older adults, a sense of social belonging, spiritual support, and opportunities for routine social participation.

The Social Network Index (SNI) is an important indicator for assessing social connectedness among older adults and comprises four dimensions: marital relationships, intimate relationships, religious and faith-based participation, and organizational involvement (23). Research has shown that faith-related and folk social activities are associated with the establishment and maintenance of social relationships and with stronger social connectedness among older adults (24).

Existing research has generated important findings on the relationship between public spaces and social connectedness among older adults; however, limited attention has been paid to culturally embedded faith-oriented public spaces and the activity pathways linking environmental perception with social connectedness. Using the folk-oriented public spaces of Licheng District in Quanzhou Ancient City as the research context, this study constructs a structural equation model of environmental perception, incorporates behavioral activities in folk public spaces as intermediate constructs, and examines the associations between environmental perception, activity participation, and social connectedness among older adults as well as the corresponding indirect associations.

The study focuses on the following issues: (1) incorporating social environmental characteristics to develop an environmental indicator system that reflects both material and social dimensions; (2) shifting the research perspective from general urban residential spaces to the folk-oriented context of a historic city; (3) employing a structural equation modeling approach to examine the pathways through which public-space environmental elements are associated with social connectedness; and (4) examining social connectedness in public spaces within specific folk-cultural contexts. Based on these considerations, the study aims to inform strategies for supporting social connectedness and age-friendly public-space planning.

This study aims to investigate how older adults’ perceptions of faith-oriented public spaces in Quanzhou Ancient City are associated with social connectedness and to test model-based indirect associations involving different forms of activity participation. Specifically, the study examines five dimensions of environmental perception—activity-oriented, landscape-oriented, accessibility, communicative, and faith-oriented elements—and three categories of activity participation—faith-based folk activities, non-faith folk activities, and non-folk activities. It hypothesizes that perceived public-space environmental elements are positively associated with social connectedness among older adults (H1), that these perceptions are positively associated with older adults’ activity participation (H2), that participation in faith-based folk activities, non-faith folk activities, and non-folk activities is associated with social connectedness (H3), and the model includes statistically testable indirect associations linking environmental perception, these forms of activity participation, and social connectedness (H4). Using pooled data from two repeated cross-sectional survey waves and adjusting for relevant sociodemographic covariates, this study applies structural equation modeling to examine the proposed associations among environmental perception, activity participation, and social connectedness.

## 2 Methods

### 2.1 Model construction and hypotheses

#### 2.1.1 Indicator system construction

Drawing on existing research and the characteristics of faith-oriented public spaces in Quanzhou Ancient City, this study organizes the environmental factors associated with social connectedness among older adults into five categories: faith-oriented elements, communicative elements, activity-oriented elements, landscape elements, and accessibility elements (25). Older adults’ perceptions of the spiritual environment in the ancient city are represented primarily by faith-oriented and communicative elements. Based on previous research and field investigations, four indicators were used to assess the spiritual environmental elements of public spaces in Quanzhou Ancient City: site-based cultural identity, social interaction, faith-based belonging, and activity participation. In this study, spiritual environmental elements refer to the socio-cultural and psychological meanings that older adults attach to public spaces, rather than to religious symbols alone, reflecting place identity, social belonging, and community connection. This definition is consistent with Kim and Kaplan’s (26) discussion of associations among physical environments, place identity, community attachment, social interaction, and shared everyday use. The perceived physical environment encompasses activity support, landscape characteristics, and accessibility, and perceptions of safe, comfortable activity spaces may be associated with greater activity participation among older adults. Considering the alternating folk-traditional activities regularly held in the ancient city, indicators representing activity-oriented elements were selected, including the diversity of public activity spaces, the inclusiveness of these spaces, and the adequacy of activity-related facilities (6). Convenient access is associated with older adults’ engagement with activity sites. Accordingly, indicators for accessibility-oriented elements include the recognizability of public spaces, the ease of walking to these spaces, and the degree of connectivity between public spaces and surrounding spaces (27).

Older adults’ social behavior and activities are closely related to their level of social connectedness. Based on a statistical analysis of field survey results, social activities among older adults in the public spaces of Quanzhou’s historic urban area can be classified into three types: faith-based folk activities, non-faith folk activities, and non-folk activities, encompassing nine major forms. This classification reflects both the cultural characteristics of Quanzhou Ancient City and the different forms of social participation available to older adults. Specifically, faith-based folk activities include territorial processions, festival celebrations, and daily prayer rituals. Non-faith folk activities include flower-wearing practices, street parades, and lantern festivals. Lastly, non-folk activities include card and chess games, leisure exercise, and charitable medical consultations.

#### 2.1.2 Conceptual model construction

This study considers traditional folk culture in the historic urban area and examines social connectedness among older adults as the dependent construct, with older adults’ activity participation in the ancient city—namely faith-based folk activities, non-faith folk activities, and non-folk activities—included as intermediate constructs. In this study, social connectedness is conceptualized as a multidimensional construct related to older adults’ social connection characteristics, including positive affect, interaction tendency, and network richness. Based on this structure, a conceptual model of environmental perception in the folk-oriented public spaces of the ancient city was constructed (28). The model follows an environment–behavior–health analytical framework, dividing environmental perception factors of public activity spaces into physical and spiritual dimensions, which may be associated with social connectedness among older adults through both direct and indirect associations (29). The model therefore specifies statistical associations among public-space environmental capacity, social activity participation, communication, interaction, and social connectedness, including a hypothesized indirect association involving activity participation (30). Accordingly, this study incorporates older adults’ social activity participation as intermediate constructs in the hypothesized model to examine the hypothesized associations among physical/spiritual environmental perception, activity participation, and social connectedness (Figure 1).

**Figure 1:**
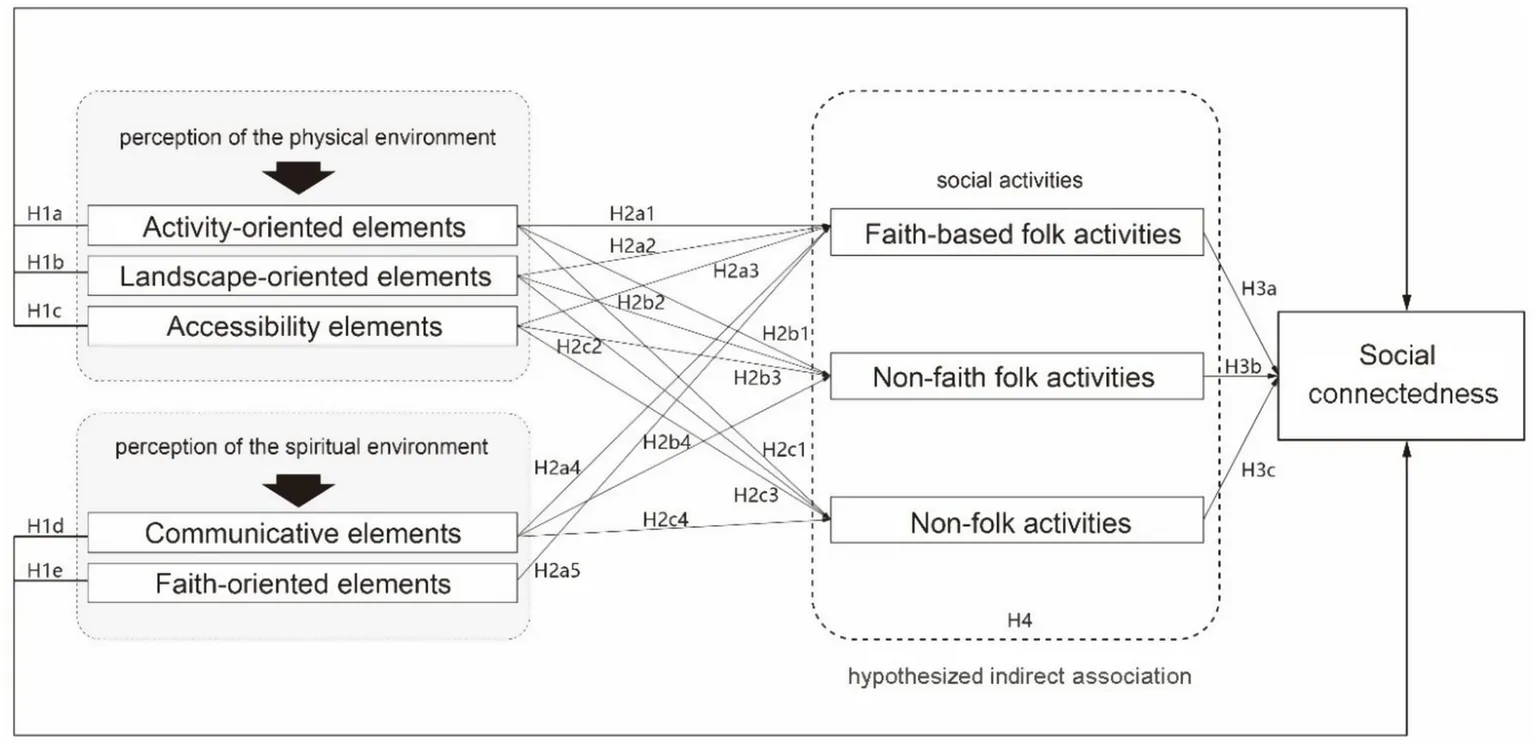
The hypothesized model.

#### 2.1.3 Research hypotheses

The corresponding path-specific hypotheses (H1a–H1e, H2a1–H2a5, H2b1–H2b4, H2c1–H2c4, H3a–H3c, and H4) are summarized in Figure 1.

### 2.2 Questionnaire design and survey

#### 2.2.1 Study area profile

In the ancient city of Quanzhou, traditional communities have persisted from the Ming and Qing dynasties to the present, and their bottom-up spatial form has developed in association with traditional folk beliefs and is characterized by a sense of belonging and a faith-based community. Within Licheng District, the Pujing temple system represents a historically embedded socio-spatial network rather than a set of isolated religious buildings. Centered on territorial temples and neighborhood-based ritual practices, it links household courtyards, shared wells, lanes, streets, and community temples into a gradual spatial hierarchy from domestic to collective public space. This layered structure is characterized by frequent everyday encounters, shared cultural practices, and stable opportunities for participation, making it a relevant setting in which to examine neighborhood familiarity, place attachment, social interaction, and social connectedness among older adults.

This locally rooted spatial structure is associated with territorial bonds, community identity, mechanisms of mutual support, and active family- and neighborhood-based networks within community public spaces (31). It therefore represents a locally distinctive, faith-oriented social environment for older adults. The “temple–community” interactive pattern reflected in the study sample therefore provides a culturally specific case for examining these associations. The location of Quanzhou Ancient City, the study boundary in Licheng District, and the principal survey sites are shown in Figure 2.

**Figure 2:**
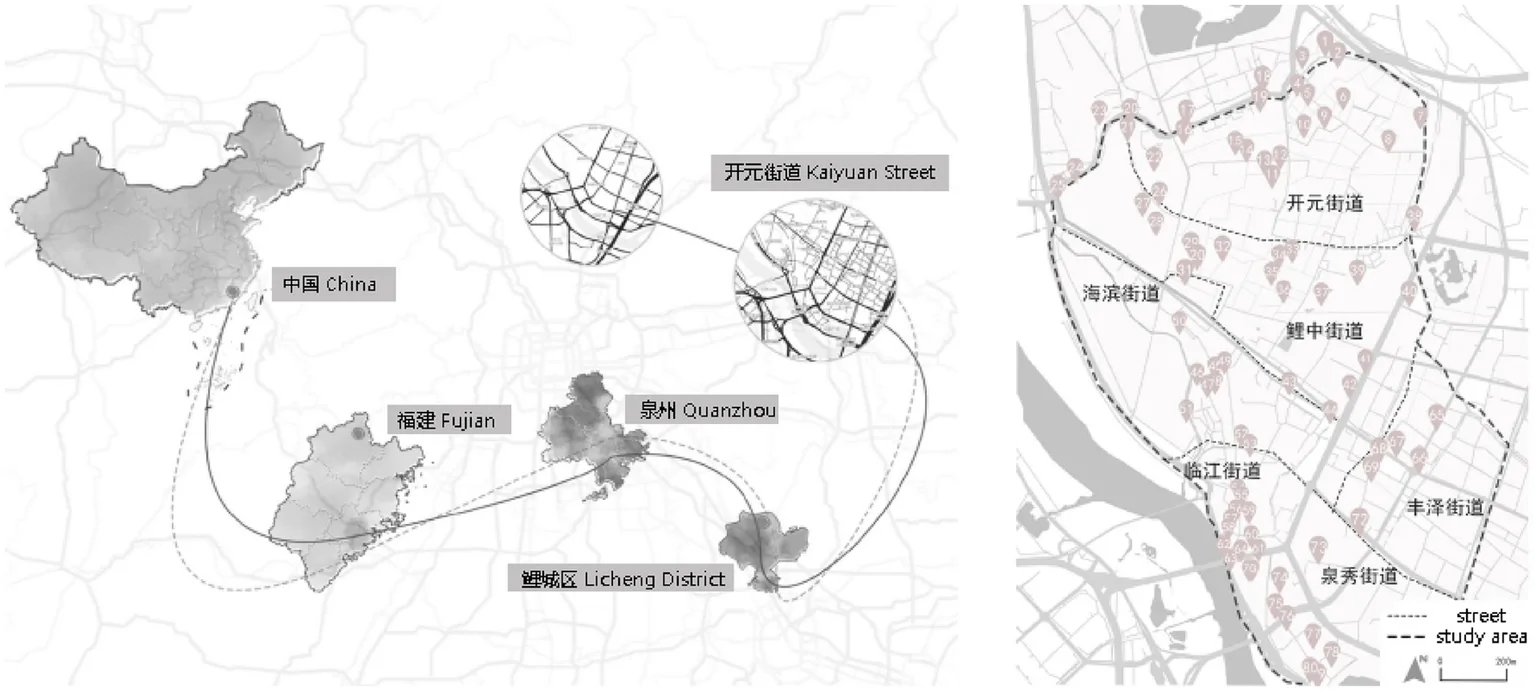
Location of Quanzhou Ancient City, the study area, and principal survey sites.

#### 2.2.2 Questionnaire design and variables

The questionnaire consisted of four sections: individual attributes of older adults, social activity participation in public spaces, social connectedness, and environmental perception of public spaces in the ancient city (3). The social connectedness measure was developed with reference to Cornwell and Waite’s multidimensional conceptualization of social disconnectedness and perceived isolation. In this study, the construct was used to capture older adults’ social connection characteristics, including positive affect, interaction tendency, and network richness, with higher scores indicating stronger social connectedness. Individual attributes included gender, age group, educational attainment, household composition, and religious affiliation. These five variables were used to characterize the sample and were included as observed covariates in the structural model because they may be associated with older adults’ opportunities for activity participation and social connection. For the social behavior section, participation levels in three types of activities—faith-based folk activities, non-faith folk activities, and non-folk activities—were specified as intermediate constructs to reflect older adults’ behavioral activity participation within the folk-cultural context of the ancient city. Environmental perception is the core measure in this study. Following multidimensional models commonly used in public-space research, environmental perception is divided into five dimensions—activity-oriented, landscape-oriented, accessibility-oriented, communicative, and faith-oriented—to reflect the two overarching categories of physical and spiritual perception (25). To clarify the distinction between environmental perception and individual behavior, Q026 was operationalized as a site-level environmental-perception item. It assessed respondents’ satisfaction with the overall level of activity participation occurring at or supported by the site (1 = very dissatisfied to 5 = very satisfied), whereas Q006–Q014 measured respondents’ own frequency of participation in nine specific activities (1 = never to 5 = always). Thus, Q026 represented an appraisal of the site’s participatory social environment rather than the respondent’s individual activity behavior. The environmental-perception items were adapted from established studies of public-space evaluation and age-friendly environments (6, 25–27), whereas the activity-participation items were developed from field observations and the locally identified classification of faith-based folk, non-faith folk, and non-folk activities. The social-connectedness items were developed as a study-specific composite to assess positive affect, social-engagement tendency, and interpersonal-network richness; they were not taken directly from, and should not be interpreted as, the Social Network Index (SNI) or the six-item Lubben Social Network Scale (LSNS-6). Before the main surveys, the item wording was reviewed by the research team and trained field investigators to improve clarity and cultural appropriateness; however, no separate formal pilot study or quantified expert content-validity assessment was conducted. The same finalized questionnaire, item wording, response options, scoring procedures, and administration protocol were used in both the October 2020 and January 2024 surveys. Item-specific wording, response anchors, scoring directions, and source or adaptation information are provided in Supplementary Table S1.

#### 2.2.3 Data sources

Two rounds of paper-based questionnaire surveys were conducted in October 2020 and January 2024 in the historic urban area of Quanzhou, China. The two waves comprised independent repeated cross-sectional samples rather than repeated observations of the same individuals. In both rounds, respondents were recruited on site from comparable neighborhood public spaces and residential areas using the same procedures. Eligible participants were community-dwelling adults aged >55 years (i.e., ≥56 years) who had resided in the study area for at least 10 years and were able to understand and complete the questionnaire. The age threshold was established before data collection to include residents entering the locally observed retirement-transition period, which is concentrated in the late 50s and around age 60. This transition is relevant because retirement may be accompanied by changes in daily activity participation and the use of neighborhood public spaces. Individuals with severe cognitive or communication impairments that prevented informed consent or questionnaire completion were excluded; trained investigators provided neutral clarification when needed.

The study protocol and the research activities reported in this manuscript were reviewed and approved by the Research Office of Xiamen University Tan Kah Kee College (Approval No. LLSC-R2026003). The ethical review covers the research procedures and data collection reported in the present study as part of the ongoing research project. All participants received oral and written information about the purpose and procedures of the study and provided written informed consent before participation. Participation was voluntary, the questionnaires were anonymous, no directly identifying information was collected, and the data were analyzed in de-identified form. Participants were informed that they could decline to answer any question or withdraw without consequence.

A total of 620 questionnaires with complete data were retained for analysis, including 333 collected in October 2020 and 287 collected in January 2024. No item-level missing data remained, and no imputation was performed. Because the total number of eligible individuals approached was not systematically recorded, a conventional response rate could not be calculated.

### 2.3 Analytical methods

This study examined the indirect associations involving older adults’ social activity participation between perceived environmental factors of faith-oriented public spaces and social connectedness among older adults. To accurately measure the relationships among variables and verify the hypothesized pathways, structural equation modeling (SEM) was employed for the empirical analysis (32). Maximum likelihood estimation was applied in AMOS 28, treating the Likert-scale variables as approximately continuous after assessing their distributional characteristics. The SEM enables the simultaneous estimation of direct and indirect statistical associations among perceived environmental characteristics, activity participation, and social connectedness. Model construction and visualization were conducted using AMOS 28, whereas data analysis and reliability and validity testing were performed using SPSS 22 to ensure the robustness and scientific rigor of the findings. Demographic characteristics were examined descriptively and were entered as observed exogenous covariates predicting the three activity factors and social connectedness. Preliminary analyses were conducted in SPSS 22. Between-wave differences in categorical participant characteristics were assessed using chi-square tests, whereas differences in continuous construct scores were assessed using independent-samples *t* tests. Multicollinearity was evaluated using variance inflation factors (VIFs), and common-method bias was assessed using an unrotated single-factor test. Multivariate normality was examined using Mardia’s multivariate kurtosis test. Because the data departed from multivariate normality, maximum likelihood estimation was retained in AMOS 28, while bootstrap procedures with 5,000 resamples were used to obtain corrected standard errors and bias-corrected 95% confidence intervals for indirect associations. Each latent construct was scaled by fixing one factor loading to 1.0. Because the communicative and faith-oriented constructs were each represented by two indicators, their measurement results were interpreted cautiously despite their high standardized loadings and acceptable composite reliability and average variance extracted values.

To evaluate whether pooling the two repeated cross-sectional samples was statistically defensible, multigroup analyses were conducted across the October 2020 (T1) and January 2024 (T2) samples. Configural invariance was evaluated using an unconstrained model, and metric invariance was tested by constraining the factor loadings to equality across survey waves. Structural stability was subsequently examined by additionally constraining the structural regression paths. Invariance was considered supported when the chi-square difference test was nonsignificant and the absolute change in CFI was below 0.010. The structural model was also re-estimated separately in T1 and T2, and corresponding path coefficients were formally compared across waves.

## 3 Data analysis

### 3.1 Sample characteristics and preliminary analyses

The pooled sample included 306 men (49.35%) and 314 women (50.65%), and 349 participants (56.29%) reported a religious or folk-belief affiliation. Participant characteristics for the pooled sample and by survey wave are presented in Table 1. Chi-square tests showed no statistically significant between-wave differences in gender (*χ*^2^ = 1.941, *p* = 0.164), age group (*χ*^2^ = 2.385, *p* = 0.665), educational attainment (*χ*^2^ = 2.545, *p* = 0.467), household composition (*χ*^2^ = 2.322, *p* = 0.677), or religious affiliation (*χ*^2^ = 0.521, *p* = 0.470). These results indicated that the sociodemographic composition of the two repeated cross-sectional samples was broadly comparable (Table 1).

**Table 1:** Participant characteristics in the pooled sample and by survey wave.

Variable	Category	Pooled (*n* = 620)	T1 (*n* = 333)	T2 (*n* = 287)	*χ^2^*	*p*
Gender	Male	306 (49.35%)	173 (52.0%)	133 (46.3%)	1.941	0.164
	Female	314 (50.65%)	160 (48.0%)	154 (53.7%)		
Age	>75	65 (10.48%)	34 (10.2%)	31 (10.8%)	2.385	0.665
	71–75	67 (10.81%)	41 (12.3%)	26 (9.1%)		
	66–70	179 (28.87%)	99 (29.7%)	80 (27.9%)		
	61–65	178 (28.71%)	92 (27.6%)	86 (30.0%)		
	56–60	131 (21.13%)	67 (20.1%)	64 (22.3%)		
Educational attainment	Primary school or below	82 (13.23%)	43 (12.9%)	39 (13.6%)	2.545	0.467
	Junior high school	211 (34.03%)	108 (32.4%)	103 (35.9%)		
	High school or technical secondary school	219 (35.32%)	127 (38.1%)	92 (32.1%)		
	Bachelor’s degree or above	108 (17.42%)	55 (16.5%)	53 (18.5%)		
Household composition	Living alone	34 (5.48%)	19 (5.7%)	15 (5.2%)	2.322	0.677
	With spouse	131 (21.13%)	67 (20.1%)	64 (22.3%)		
	With children	193 (31.13%)	103 (30.9%)	90 (31.4%)		
	With grandchildren	180 (29.03%)	94 (28.2%)	86 (30.0%)		
	Three generations living together	82 (13.23%)	50 (15.0%)	32 (11.1%)		
Faith	No faith	271 (43.71%)	150 (45.0%)	121 (42.2%)	0.521	0.470
	Has faith	349 (56.29%)	183 (55.0%)	166 (57.8%)		

Independent-samples t tests further showed no statistically significant between-wave differences in any of the study constructs. In particular, social connectedness scores were comparable between October 2020 (M = 3.04, SD = 0.98) and January 2024 (M = 3.08, SD = 0.94; *t* = −0.569, *p* = 0.570). The *p*-values for the remaining constructs ranged from 0.190 to 0.989, providing additional support for pooling the two survey waves (Table 2).

**Table 2:** Descriptive statistics and between-wave comparisons of the study constructs.

Construct	T1 (*n* = 333)	T2 (*n* = 287)	*t*	*p*	Skewness	Kurtosis
Social connectedness	3.04 ± 0.98	3.08 ± 0.94	−0.569	0.570	−0.15	−0.58
Faith-based folk activities	3.24 ± 1.01	3.22 ± 0.96	0.230	0.818	−0.40	−0.19
Non-faith folk activities	3.28 ± 1.05	3.28 ± 1.02	−0.014	0.989	−0.41	−0.34
Non-folk activities	3.29 ± 0.96	3.32 ± 0.93	−0.370	0.711	−0.30	−0.15
Activity-oriented elements	3.23 ± 1.05	3.25 ± 1.01	−0.327	0.744	−0.43	−0.33
Landscape-oriented elements	3.22 ± 0.94	3.21 ± 0.93	0.150	0.881	−0.38	0.02
Accessibility elements	2.84 ± 0.93	2.94 ± 0.95	−1.311	0.190	0.50	−0.41
Communicative elements	3.17 ± 1.03	3.25 ± 1.12	−0.945	0.345	−0.30	−0.49
Faith-oriented elements	3.23 ± 1.13	3.25 ± 1.07	−0.217	0.828	−0.39	−0.47

The questionnaire first underwent reliability and validity testing, as well as an assessment of common method bias, followed by confirmatory factor analysis to examine convergent and discriminant validity. Overall reliability was assessed using internal consistency measures, whereas exploratory dimensionality was examined using principal component analysis with Varimax rotation and Kaiser normalization; components with eigenvalues greater than 1.0 were retained. Confirmatory factor analysis employed goodness-of-fit indices, standardized factor loadings (SFL), composite reliability (CR), and average variance extracted (AVE) to jointly assess structural validity, convergent validity, and discriminant validity (32). In this study, the independent, mediating, and dependent variables exhibited certain correlations while remaining distinguishable; therefore, confirmatory factor analysis was conducted without separating them into different models. The data were analyzed using AMOS 28 to perform the confirmatory factor analysis.

In the overall reliability and validity analysis of the questionnaire, the Cronbach’s alpha coefficient was 0.902, which was well above the threshold of 0.7, and this value decreased or remained unchanged when any item was deleted, indicating that the requirement was met. The KMO value of 0.858 likewise exceeded the threshold of 0.7, and Bartlett’s test of sphericity yielded a statistic of 9800.481 with a significance level below 0.001 (reference value < 0.05), demonstrating that the variables were suitable for factor analysis. The analysis retained nine components, corresponding to the nine prespecified first-order constructs and explaining 80.829% of the total variance. In the rotated solution, all items loaded most strongly on their corresponding components, with primary loadings ranging from 0.704 to 0.918; rotation converged after seven iterations (Supplementary Table S2). Construct-specific Cronbach’s alpha coefficients ranged from 0.813 to 0.910 across the nine first-order constructs. The alpha coefficients were 0.861 for faith-based folk activities, 0.910 for non-faith folk activities, 0.847 for non-folk activities, 0.910 for activity-oriented elements, 0.907 for landscape-oriented elements, 0.836 for accessibility-oriented elements, 0.878 for communicative elements, 0.892 for faith-oriented elements, and 0.813 for social connectedness, indicating acceptable-to-high internal consistency. Because the principal variables were collected using the same self-report questionnaire, an unrotated single-factor test was conducted to assess common-method bias. The first factor had an eigenvalue of 7.696 and explained 29.602% of the total variance, below the prespecified 40% threshold. Thus, the test did not indicate a dominant single-factor problem, although residual common-method bias cannot be completely excluded. Univariate skewness and kurtosis critical ratios ranged from −4.391 to 4.693 and from −4.625 to −1.199, respectively, indicating no severe univariate nonnormality. However, Mardia’s multivariate kurtosis coefficient was 193.457, with a critical ratio of 63.12, indicating departure from multivariate normality. VIF values ranged from 1.142 to 1.529, well below the threshold of 5, suggesting that multicollinearity was unlikely to distort the structural path estimates.

According to the model estimation results, the nine-construct CFA measurement model showed good fit (*χ*^2^/df = 1.099, RMR = 0.027, GFI = 0.966, NFI = 0.971, RFI = 0.964, IFI = 0.997, TLI = 0.997, CFI = 0.997, and RMSEA = 0.013; Table 3). Taken together, these indices indicate that the measurement model showed an acceptable-to-good fit with the observed data, as shown in Table 3.

**Table 3:** Confirmatory factor analysis—goodness-of-fit indices.

Fit index (abbrev.)	Recommended threshold	Model value	Meets criterion
Chi-square/df (*χ*^2^/df)	1–3	1.099	Yes
Root mean square residual (RMR)	<0.08	0.027	Yes
Goodness of Fit Index (GFI)	>0.90	0.966	Yes
Normed Fit Index (NFI)	>0.90	0.971	Yes
Relative Fit Index (RFI)	>0.90	0.964	Yes
Incremental Fit Index (IFI)	>0.90	0.997	Yes
Tucker–Lewis Index (TLI)	>0.90	0.997	Yes
Comparative Fit Index (CFI)	>0.90	0.997	Yes
Root mean square error of approximation (RMSEA)	<0.08	0.013	Yes

Convergent validity was first assessed using standardized factor loadings, composite reliability, and average variance extracted. The results showed that all indicators corresponding to the nine latent variables passed the significance test (*p* < 0.001), with standardized factor loadings all exceeding 0.6 (ranging from 0.704 to 0.957). Standardized factor loadings ranged from 0.704 to 0.957, composite reliability values ranged from 0.813 to 0.911, and AVE values ranged from 0.594 to 0.811. All CR values exceeded the recommended threshold of 0.70, and all AVE values exceeded the conventional threshold of 0.50, supporting convergent validity (Table 4); the full measurement model is presented in Supplementary Figure S1. Discriminant validity was assessed using the Fornell–Larcker criterion (33). The square roots of the AVE values ranged from 0.771 to 0.901 and exceeded the corresponding interconstruct correlations, which ranged from 0.108 to 0.582. These results supported the discriminant validity of the nine-construct measurement model (Table 5).

**Table 4:** Confirmatory factor analysis—and convergent validity.

First-order construct	Item	Estimate	S.E.	C.R.	*p*	Std estimate	CR	AVE
Faith-based folk activities	Q006	1.000				0.789	0.862	0.677
	Q007	1.150	0.052	22.295	***	0.906		
	Q008	0.957	0.048	19.856	***	0.766		
Non-faith folk activities	Q009	1.000				0.863	0.911	0.775
	Q010	0.970	0.037	25.926	***	0.815		
	Q011	1.128	0.035	31.872	***	0.957		
Non-folk activities	Q012	1.000				0.798	0.848	0.651
	Q013	1.062	0.050	21.132	***	0.851		
	Q014	0.969	0.050	19.467	***	0.770		
Activity-oriented elements	Q015	1.049	0.040	26.551	***	0.857	0.911	0.774
	Q016	1.138	0.039	29.269	***	0.939		
	Q017	1.000				0.840		
Landscape-oriented elements	Q018	0.977	0.044	22.396	***	0.808	0.908	0.711
	Q019	1.000				0.801		
	Q020	1.095	0.043	25.588	***	0.896		
	Q021	1.055	0.043	24.489	***	0.864		
Accessibility elements	Q022	1.033	0.050	20.533	***	0.855	0.839	0.636
	Q023	0.849	0.048	17.768	***	0.704		
	Q024	1.000				0.825		
Communicative elements	Q025	0.969	0.072	13.480	***	0.880	0.878	0.783
	Q026	1.000				0.890		
Faith-oriented elements	Q027	0.895	0.055	16.407	***	0.845	0.895	0.811
	Q028	1.000				0.953		
Social connectedness	Q029	1.079	0.065	16.553	***	0.753	0.813	0.594
	Q030	1.202	0.067	17.862	***	0.848		
	Q031	1.000				0.704		

*** p < 0.001; ** p < 0.01; * p < 0.05.

**Table 5:** Discriminant validity of the measurement constructs using the Fornell–Larcker criterion.

Construct	1	2	3	4	5	6	7	8	9
1. Faith-based folk activities	0.823								
2. Non-faith folk activities	0.419**	0.880							
3. Non-folk activities	0.441**	0.422**	0.807						
4. Activity-oriented elements	0.316**	0.237**	0.301**	0.880					
5. Landscape-oriented elements	0.204**	0.172**	0.451**	0.298**	0.843				
6. Accessibility elements	0.172**	0.295**	0.328**	0.210**	0.418**	0.797			
7. Communicative elements	0.108*	0.226**	0.317**	0.211**	0.273**	0.225**	0.885		
8. Faith-oriented elements	0.264**	0.189**	0.239**	0.431**	0.248**	0.190**	0.147**	0.901	
9. Social connectedness	0.486**	0.466**	0.582**	0.417**	0.472**	0.448**	0.369**	0.360**	0.771

Diagonal entries are the square roots of AVE; off-diagonal entries are interconstruct correlations. ** *p* < 0.01; * *p* < 0.05.

### 3.2 Survey-wave measurement invariance and robustness analyses

The unconstrained multigroup model showed acceptable fit across the two survey waves (*χ*^2^ = 875.168, df = 705, *χ*^2^/df = 1.241, RMR = 0.058, GFI = 0.918, NFI = 0.919, CFI = 0.983, and RMSEA = 0.020). Constraining the factor loadings to equality did not significantly reduce model fit (*χ*^2^ = 890.690, df = 722; Δ*χ*^2^ = 15.522, Δdf = 17, *p* = 0.558; ΔCFI = 0.000), supporting metric invariance across T1 and T2. A model that additionally constrained the structural weights also showed acceptable fit (*χ*^2^ = 935.427, df = 765, *χ*^2^/df = 1.223, CFI = 0.983, and RMSEA = 0.019) and did not differ significantly from the metric-invariance model (Δ*χ*^2^ = 44.737, Δdf = 43, *p* = 0.399; ΔCFI = 0.000). These findings support the comparability of the factor loadings and structural associations across survey waves. Because scalar invariance was not tested, these findings support comparisons of structural associations, but not latent means (Supplementary Tables S3A,S3B).

Wave-stratified sensitivity analyses showed that none of the 21 focal path coefficients differed significantly between T1 and T2, with between-wave *p* values ranging from 0.053 to 0.874 (Supplementary Table S4). These results indicate that the pooled findings were similar across the two survey waves and supported use of the pooled sample.

### 3.3 Path analysis

The covariate-adjusted structural model showed acceptable fit (*χ*^2^/df = 1.099, CFI = 0.997, TLI = 0.997, RMSEA = 0.013). After adjustment for gender, age, educational attainment, household composition, and religious affiliation, all five dimensions of public-space environmental perception were positively associated with social connectedness. The standardized coefficients were 0.100 for activity-oriented elements (*p* = 0.017), 0.157 for landscape-oriented elements (*p* < 0.001), 0.169 for accessibility-oriented elements (*p* < 0.001), 0.125 for communicative elements (*p* = 0.001), and 0.091 for faith-oriented elements (*p* = 0.022). Accessibility-oriented perception showed the largest direct association with social connectedness, although the magnitudes of the five direct associations were generally modest.

Environmental perceptions showed activity-specific associations. Faith-based folk participation was positively associated with activity-oriented elements (*β* = 0.194, *p* < 0.001) and faith-oriented elements (*β* = 0.112, *p* = 0.017), whereas its associations with landscape-oriented (*β* = 0.058, *p* = 0.229), accessibility-oriented (*β* = 0.072, *p* = 0.137), and communicative elements (*β* = −0.011, *p* = 0.801) were not significant. Non-faith folk participation was positively associated with activity-oriented (*β* = 0.141, *p* = 0.001), accessibility-oriented (*β* = 0.233, *p* < 0.001), and communicative elements (*β* = 0.127, *p* = 0.004), but not with landscape-oriented elements (*β* = −0.019, *p* = 0.684). Non-folk participation was positively associated with activity-oriented (*β* = 0.115, *p* = 0.006), landscape-oriented (*β* = 0.288, *p* < 0.001), accessibility-oriented (*β* = 0.132, *p* = 0.004), and communicative elements (*β* = 0.152, *p* < 0.001).

All three activity categories were positively associated with social connectedness: faith-based folk activities (*β* = 0.169, *p* < 0.001), non-faith folk activities (*β* = 0.145, *p* < 0.001), and non-folk activities (*β* = 0.180, *p* < 0.001). Among the covariates, household composition was positively associated with social connectedness (*β* = 0.149, *p* < 0.001), whereas gender, age, educational attainment, and religious affiliation showed no significant direct associations. The complete path estimates are reported in Table 6, and the estimated structural model with unstandardized path coefficients is shown in Figure 3.

**Table 6:** Covariate-adjusted structural path estimates.

Path	Estimate	STD Estimate	S.E.	C.R.	*p*
Faith-based folk activities ← activity-oriented elements	0.186	0.194	0.046	4.035	***
Non-faith folk activities ← activity-oriented elements	0.145	0.141	0.044	3.272	0.001
Non-faith folk activities ← landscape-oriented elements	−0.021	−0.019	0.052	−0.407	0.684
Non-faith folk activities ← accessibility elements	0.247	0.233	0.051	4.850	***
Non-faith folk activities ← communicative elements	0.119	0.127	0.041	2.879	0.004
Non-folk activities ← communicative elements	0.129	0.152	0.036	3.547	***
Non-folk activities ← activity-oriented elements	0.107	0.115	0.039	2.749	0.006
Non-folk activities ← landscape-oriented elements	0.288	0.288	0.047	6.103	***
Non-folk activities ← accessibility elements	0.127	0.132	0.044	2.874	0.004
Faith-based folk activities ← faith-oriented elements	0.090	0.112	0.038	2.388	0.017
Faith-based folk activities ← communicative elements	−0.010	−0.011	0.039	−0.252	0.801
Faith-based folk activities ← accessibility elements	0.071	0.072	0.048	1.488	0.137
Faith-based folk activities ← landscape-oriented elements	0.060	0.058	0.050	1.204	0.229
Social connectedness ← activity-oriented elements	0.084	0.100	0.035	2.379	0.017
Social connectedness ← landscape-oriented elements	0.142	0.157	0.040	3.589	***
Social connectedness ← accessibility elements	0.147	0.169	0.038	3.883	***
Social connectedness ← communicative elements	0.096	0.125	0.030	3.177	0.001
Social connectedness ← faith-oriented elements	0.064	0.091	0.028	2.286	0.022
Social connectedness ← faith-based folk activities	0.148	0.169	0.036	4.122	***
Social connectedness ← non-faith folk activities	0.119	0.145	0.031	3.783	***
Social connectedness ← non-folk activities	0.163	0.180	0.042	3.843	***
Faith-based folk activities ← gender	0.001	0.001	0.069	0.020	0.984
Faith-based folk activities ← age	0.072	0.099	0.028	2.544	0.011
Faith-based folk activities ← educational attainment	0.082	0.085	0.038	2.174	0.030
Faith-based folk activities ← household composition	0.195	0.239	0.034	5.819	***
Faith-based folk activities ← faith	0.114	0.063	0.070	1.618	0.106
Non-faith folk activities ← gender	0.022	0.012	0.073	0.306	0.760
Non-faith folk activities ← age	0.046	0.059	0.030	1.538	0.124
Non-faith folk activities ← educational attainment	0.055	0.053	0.040	1.384	0.166
Non-faith folk activities ← household composition	0.140	0.161	0.035	4.054	***
Non-faith folk activities ← faith	0.117	0.061	0.074	1.584	0.113
Non-folk activities ← gender	0.050	0.029	0.064	0.790	0.430
Non-folk activities ← age	0.035	0.049	0.026	1.324	0.186
Non-folk activities ← educational attainment	0.084	0.090	0.035	2.433	0.015
Non-folk activities ← household composition	0.166	0.210	0.031	5.414	***
Non-folk activities ← faith	0.138	0.079	0.065	2.132	0.033
Social connectedness ← gender	−0.007	−0.004	0.051	−0.130	0.897
Social connectedness ← age	0.004	0.007	0.021	0.205	0.837
Social connectedness ← educational attainment	0.042	0.050	0.028	1.493	0.136
Social connectedness ← household composition	0.106	0.149	0.027	3.942	***
Social connectedness ← faith	0.071	0.045	0.053	1.356	0.175

*** *p* < 0.001; ** *p* < 0.01; * *p* < 0.05.

**Figure 3:**
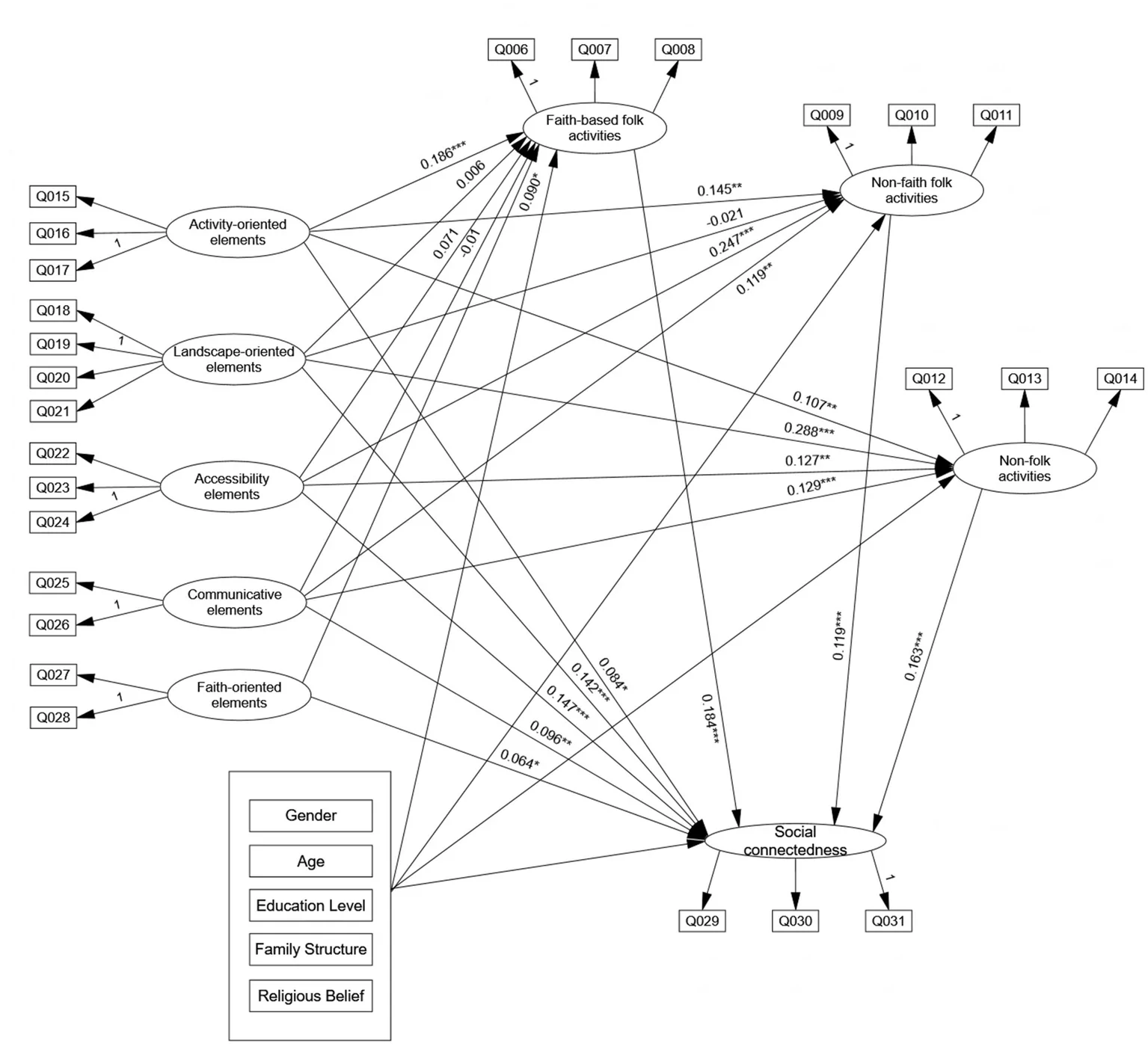
Covariate-adjusted structural model showing unstandardized path coefficients among perceived public-space environmental elements, activity participation, and social connectedness. Note. *** *p* < 0.001; ** *p* < 0.01; * *p* < 0.05.

### 3.4 Indirect-association analysis

Indirect associations were assessed using 5,000 bootstrap resamples and bias-corrected 95% confidence intervals. An indirect association was considered statistically significant when its confidence interval did not include zero. The bootstrap estimates are reported in Table 7.

**Table 7:** Bootstrap estimates of direct, specific indirect, total indirect, and total associations with bias-corrected 95% confidence intervals.

Association type	Path	Estimate	95% lower	95% upper	*p*	Proportion of the total association
Total association	Activity-oriented elements → … → social connectedness	0.146	0.071	0.226	<0.001	-
	Landscape-oriented elements → … → social connectedness	0.195	0.099	0.300	<0.001	-
	Accessibility elements → … → social connectedness	0.207	0.122	0.301	<0.001	-
	Communicative elements → … → social connectedness	0.129	0.057	0.208	0.001	-
	Faith-oriented elements → … → social connectedness	0.078	0.017	0.148	0.010	-
Direct association	Activity-oriented elements → social connectedness	0.084	0.016	0.161	0.017	57.5%
	Landscape-oriented elements → social connectedness	0.142	0.046	0.241	0.004	72.8%
	Accessibility elements → social connectedness	0.147	0.068	0.234	<0.001	71.0%
	Communicative elements → social connectedness	0.096	0.029	0.169	0.006	74.4%
	Faith-oriented elements → social connectedness	0.064	0.004	0.131	0.036	82.1%
Specific indirect association	Activity-oriented elements → faith-based folk activities → social connectedness	0.028	0.011	0.055	0.001	19.2%
	Landscape-oriented elements → faith-based folk activities → social connectedness	0.009	−0.007	0.032	0.239	-
	Accessibility elements → faith-based folk activities → social connectedness	0.011	−0.003	0.032	0.126	-
	Communicative elements → faith-based folk activities → social connectedness	−0.001	−0.018	0.012	0.769	-
	Faith-oriented elements → faith-based folk activities → social connectedness	0.013	0.003	0.032	0.013	16.7%
	Activity-oriented elements → non-faith folk activities → social connectedness	0.017	0.004	0.041	0.003	11.6%
	Landscape-oriented elements → non-faith folk activities → social connectedness	−0.003	−0.020	0.013	0.702	-
	Accessibility elements → non-faith folk activities → social connectedness	0.029	0.010	0.059	0.002	14.0%
	Communicative elements → non-faith folk activities → social connectedness	0.014	0.003	0.034	0.010	10.9%
	Activity-oriented elements → non-folk activities → social connectedness	0.017	0.004	0.04	0.006	11.6%
	Landscape-oriented elements → non-folk activities → social connectedness	0.047	0.018	0.089	0.001	24.1%
	Accessibility elements → non-folk activities → social connectedness	0.021	0.005	0.049	0.006	10.1%
	Communicative elements → non-folk activities → social connectedness	0.021	0.006	0.047	0.004	16.3%
Total indirect association	Activity-oriented elements → … → social connectedness	0.062	0.034	0.099	<0.001	42.5%
	Landscape-oriented elements → … → social connectedness	0.053	0.007	0.107	0.023	27.2%
	Accessibility elements → … → social connectedness	0.061	0.023	0.108	0.001	29.5%
	Communicative elements → … → social connectedness	0.034	0.003	0.068	0.036	26.4%
	Faith-oriented elements → … → social connectedness	0.013	0.003	0.032	0.013	16.7%

Activity-oriented perception showed significant specific indirect associations with social connectedness through faith-based folk activities (B = 0.028, 95% CI [0.011, 0.055]), non-faith folk activities (B = 0.017, 95% CI [0.004, 0.041]), and non-folk activities (B = 0.017, 95% CI [0.004, 0.040]). Landscape-oriented perception showed a significant indirect association only through non-folk activities (B = 0.047, 95% CI [0.018, 0.089]); the confidence intervals for faith-based and non-faith folk activities included zero.

Accessibility-oriented perception showed significant indirect associations through non-faith folk activities (B = 0.029, 95% CI [0.010, 0.059]) and non-folk activities (B = 0.021, 95% CI [0.005, 0.049]), but not through faith-based folk activities (B = 0.011, 95% CI [−0.003, 0.032]). Communicative perception showed significant indirect associations through non-faith folk activities (B = 0.014, 95% CI [0.003, 0.034]) and non-folk activities (B = 0.021, 95% CI [0.006, 0.047]), whereas the indirect association through faith-based folk activities was not significant (B = −0.001, 95% CI [−0.018, 0.012]). Faith-oriented perception showed a significant indirect association through faith-based folk activities (B = 0.013, 95% CI [0.003, 0.032]).

The total indirect associations were significant for activity-oriented (B = 0.062, 95% CI [0.034, 0.099]), landscape-oriented (B = 0.053, 95% CI [0.007, 0.107]), accessibility-oriented (B = 0.061, 95% CI [0.023, 0.108]), communicative (B = 0.034, 95% CI [0.003, 0.068]), and faith-oriented perception (B = 0.013, 95% CI [0.003, 0.032]). Accordingly, H1 and H3 were supported, H2 was partially supported, and H4 received pathway-specific support. These results indicate that the indirect associations were pathway-specific rather than uniform across activity types.

## 4 Conclusion and discussion

The present study produced five key findings. First, activity-oriented perception was associated with social connectedness both directly and indirectly through all three activity categories. Second, the indirect association for landscape-oriented perception occurred specifically through non-folk activities. Third, accessibility-oriented perception showed indirect associations through non-faith folk and non-folk activities, but not through faith-based folk activities. Fourth, communicative perception showed the same two activity-specific pathways. Fifth, faith-oriented perception was associated with social connectedness directly and indirectly through faith-based folk activities. These results indicate pathway-specific associations rather than uniform indirect associations across all activity types.

Several hypothesized associations with activity participation were not statistically significant. Landscape-, accessibility-, and communicative-element perceptions were not associated with faith-based folk participation, and landscape-oriented perceptions were not associated with non-faith folk participation. One possible explanation is that faith-based activities in the pujing context are organized mainly through ritual schedules and established community networks, whereas non-faith and non-folk activities depend more directly on everyday spatial conditions. This interpretation should be examined in future research.

Taken together, older adults’ perceptions of public spaces were associated with their participation patterns and social connectedness. Although the design does not establish causality, the findings may inform age-friendly renewal of historic districts by drawing attention to both material attributes (activity support, landscape, and accessibility) and socio-cultural attributes (communicative and faith-oriented elements) of public spaces.

Several limitations should be acknowledged. First, the repeated cross-sectional design precludes causal inference and does not permit analysis of within-person change. Although between-wave comparisons and metric-invariance tests supported pooling the samples for analysis of structural associations, scalar invariance was not tested; latent means should therefore not be compared across waves. Second, on-site convenience recruitment and the absence of a recorded denominator may limit representativeness and prevented calculation of a response rate. Third, the single-city setting limits generalizability to other historical and cultural contexts. Fourth, the self-reported measures may be affected by common-method and recall bias. Finally, social connectedness was measured using a study-specific multidimensional composite rather than a standard instrument such as the LSNS-6, limiting direct comparison with studies of objective social isolation.

## Data Availability

The original contributions presented in the study are included in the article/Supplementary material, further inquiries can be directed to the corresponding author.
