# Supplementary material for: Perceived faith-oriented public-space environments, activity participation, and social connectedness among older adults in Quanzhou Ancient City
Source: Front Public Health. 2026 Sep 23;14:1892546. doi: 10.3389/fpubh.2026.1892546 (PMC13642299; doi:10.3389/fpubh.2026.1892546)
Supplement: Supplementary file 1 [file Supplementary_file_1.docx]

### Supplementary Tables

**Supplementary Table S1**

Survey questionnaire.

| Item code | Category | Indicator | Variable description |
| --- | --- | --- | --- |
| Q001 | Individual attributes | Gender | Female = 0; Male = 1 |
| Q002 |  | Age | > 75 = 1; 71–75 = 2; 66–70 = 3; 61–65 = 4; 56-60 = 5 |
| Q003 |  | Educational attainment | Primary school or below = 1; Junior high school = 2; High school or technical secondary school = 3; Bachelor’s degree or above = 4 |
| Q004 |  | Household composition | Living alone = 1; With spouse = 2; With children = 3; With grandchildren = 4; Three generations living together = 5 |
| Q005 |  | Faith | No faith = 0; Has faith = 1 |
| Q006 | Social activities | Faith-based folk activities | Procession — 1 = never participate ~ 5 = always participate |
| Q007 |  |  | Festival birthdays — 1 = never participate ~ 5 = always participate |
| Q008 |  |  | Daily blessings — 1 = never participate ~ 5 = always participate |
| Q009 |  | Non-faith folk activities | Flower-wearing (Zan Hua) — 1 = never participate ~ 5 = always participate |
| Q010 |  |  | Street parades — 1 = never participate ~ 5 = always participate |
| Q011 |  |  | Lantern fairs — 1 = never participate ~ 5 = always participate |
| Q012 |  | Non-folk activities | Board/card games (e.g., Mahjong) — 1 = never participate ~ 5 = always participate |
| Q013 |  |  | Leisure exercise — 1 = never participate ~ 5 = always participate |
| Q014 |  |  | Free clinics/charity — 1 = never participate ~ 5 = always participate |
| Q015 | Environmental perception | Activity-related factors | Diversity of activity spaces — 1 = very dissatisfied ~ 5 = very satisfied |
| Q016 |  |  | Inclusiveness of activity spaces — 1 = very dissatisfied ~ 5 = very satisfied |
| Q017 |  |  | Adequacy of activity facilities — 1 = very dissatisfied ~ 5 = very satisfied |
| Q018 |  | Landscape-related factors | Diversity of vegetation types — 1 = very dissatisfied ~ 5 = very satisfied |
| Q019 |  |  | Richness of landscape colors — 1 = very dissatisfied ~ 5 = very satisfied |
| Q020 |  |  | Vegetation coverage — 1 = very dissatisfied ~ 5 = very satisfied |
| Q021 |  |  | Landscape accessibility — 1 = very dissatisfied ~ 5 = very satisfied |
| Q022 |  | Accessibility-related factors | Spatial legibility — 1 = very dissatisfied ~ 5 = very satisfied |
| Q023 |  |  | Walking convenience — 1 = very dissatisfied ~ 5 = very satisfied |
| Q024 |  |  | Connectivity with other spaces — 1 = very dissatisfied ~ 5 = very satisfied |
| Q025 |  | Interaction-related factors | Degree of social interaction — 1 = very dissatisfied ~ 5 = very satisfied |
| Q026 |  | Perceived site-level participation atmosphere | Degree of activity participation — 1 = very dissatisfied ~ 5 = very satisfied |
| Q027 |  | Faith-related factors | Cultural identity of the site — 1 = very dissatisfied ~ 5 = very satisfied |
| Q028 |  |  | Sense of faith-based belonging — 1 = very dissatisfied ~ 5 = very satisfied |
| Q029 | Social connectedness | Positive emotions | Mean score of: hopeful about life; feel valuable; willing to learn new things; curious (1 = never ~ 5 = always) |
| Q030 |  | Positive social engagement tendency | Mean score of: willing to interact with others; willing to engage with the outside world; willing to participate in activities (1 = never ~ 5 = always) |
| Q031 |  | Rich interpersonal network | Mean score of: frequent interaction with family; frequent interaction with friends; continuously making new friends (1 = never ~ 5 = always) |

**Supplementary Table S2**
Exploratory principal component analysis: total variance explained and rotated component loadings

**Panel A**

| Component | Initial Eigenvalues | | | Extraction Sums of Squared Loadings | | |
| --- | --- | --- | --- | --- | --- | --- |
|  | Total | Variance % | Cumulative % | Total | Variance % | Cumulative % |
| 1 | 7.696 | 29.602 | 29.602 | 7.696 | 29.602 | 29.602 |
| 2 | 2.691 | 10.348 | 39.950 | 2.691 | 10.348 | 39.950 |
| 3 | 2.298 | 8.840 | 48.790 | 2.298 | 8.840 | 48.790 |
| 4 | 1.748 | 6.725 | 55.514 | 1.748 | 6.725 | 55.514 |
| 5 | 1.629 | 6.265 | 61.779 | 1.629 | 6.265 | 61.779 |
| 6 | 1.397 | 5.372 | 67.151 | 1.397 | 5.372 | 67.151 |
| 7 | 1.275 | 4.903 | 72.054 | 1.275 | 4.903 | 72.054 |
| 8 | 1.227 | 4.720 | 76.775 | 1.227 | 4.720 | 76.775 |
| 9 | 1.054 | 4.054 | 80.829 | 1.054 | 4.054 | 80.829 |
|  | | | | | | |

**Panel B**

|  | Component | | | | | | | | |
| --- | --- | --- | --- | --- | --- | --- | --- | --- | --- |
|  | 1 | 2 | 3 | 4 | 5 | 6 | 7 | 8 | 9 |
| Q006 |  |  |  | 0.821 |  |  |  |  |  |
| Q007 |  |  |  | 0.834 |  |  |  |  |  |
| Q008 |  |  |  | 0.868 |  |  |  |  |  |
| Q009 |  |  | 0.881 |  |  |  |  |  |  |
| Q010 |  |  | 0.870 |  |  |  |  |  |  |
| Q011 |  |  | 0.885 |  |  |  |  |  |  |
| Q012 |  |  |  |  | 0.808 |  |  |  |  |
| Q013 |  |  |  |  | 0.822 |  |  |  |  |
| Q014 |  |  |  |  | 0.787 |  |  |  |  |
| Q015 |  | 0.877 |  |  |  |  |  |  |  |
| Q016 |  | 0.892 |  |  |  |  |  |  |  |
| Q017 |  | 0.867 |  |  |  |  |  |  |  |
| Q018 | 0.831 |  |  |  |  |  |  |  |  |
| Q019 | 0.838 |  |  |  |  |  |  |  |  |
| Q020 | 0.858 |  |  |  |  |  |  |  |  |
| Q021 | 0.860 |  |  |  |  |  |  |  |  |
| Q022 |  |  |  |  |  | 0.844 |  |  |  |
| Q023 |  |  |  |  |  | 0.811 |  |  |  |
| Q024 |  |  |  |  |  | 0.842 |  |  |  |
| Q025 |  |  |  |  |  |  |  | 0.914 |  |
| Q026 |  |  |  |  |  |  |  | 0.914 |  |
| Q027 |  |  |  |  |  |  |  |  | 0.918 |
| Q028 |  |  |  |  |  |  |  |  | 0.898 |
| Q029 |  |  |  |  |  |  | 0.818 |  |  |
| Q030 |  |  |  |  |  |  | 0.704 |  |  |
| Q031 |  |  |  |  |  |  | 0.735 |  |  |

*Note. Extraction method: principal component analysis. Rotation method: Varimax with Kaiser normalization. Components with eigenvalues greater than 1.0 were retained. Rotation converged in seven iterations.*

**Supplementary Table S3A**
Fit indices for multigroup measurement- and structural-invariance models across the two survey waves

| Model | CMIN | DF | p | CMIN/DF | RMR | GFI | NFI | CFI | RMSEA |
| --- | --- | --- | --- | --- | --- | --- | --- | --- | --- |
| Unconstrained | 875.168 | 705.000 | < 0.001 | 1.241 | 0.058 | 0.918 | 0.919 | 0.983 | 0.020 |
| Measurement weights | 890.690 | 722.000 | < 0.001 | 1.234 | 0.059 | 0.916 | 0.917 | 0.983 | 0.019 |
| Structural weights | 935.427 | 765.000 | < 0.001 | 1.223 | 0.066 | 0.913 | 0.913 | 0.983 | 0.019 |

**Supplementary Table S3B**
Changes in model-fit indices for measurement- and structural-invariance tests across the two survey waves

| Model | Δ CMIN | Δ DF | p | Δ GFI | Δ NFI | Δ RFI | Δ IFI | Δ TLI | Δ CFI |
| --- | --- | --- | --- | --- | --- | --- | --- | --- | --- |
| Measurement weights | 15.522 | 17 | 0.558 | -0.002 | -0.002 | 0.000 | -0.001 | 0.000 | 0.000 |
| Structural weights | 44.737 | 43 | 0.399 | -0.003 | -0.004 | 0.000 | -0.001 | 0.000 | 0.000 |

**Supplementary Table S4**
Structural path estimates by survey wave and tests of differences between October 2020 and January 2024

| Path | T1/Oct 2020 | | | | T2/Jan 2024 | | | | z | p |
| --- | --- | --- | --- | --- | --- | --- | --- | --- | --- | --- |
|  | Estimate | S.E. | C.R. | P | Estimate | S.E. | C.R. | P |  |  |
| Faith-based folk activities <--- Activity-oriented elements | 0.260 | 0.062 | 4.201 | *** | 0.134 | 0.043 | 3.155 | 0.002 | 1.670 | 0.095 |
| Non-faith folk activities <--- Activity-oriented elements | 0.164 | 0.061 | 2.668 | 0.008 | 0.040 | 0.036 | 1.097 | 0.273 | 1.751 | 0.080 |
| Non-faith folk activities <---Landscape-oriented elements | 0.047 | 0.068 | 0.698 | 0.485 | -0.128 | 0.081 | -1.571 | 0.116 | 1.655 | 0.098 |
| Non-faith folk activities <---Accessibility elements | 0.218 | 0.064 | 3.403 | *** | 0.257 | 0.081 | 3.188 | 0.001 | -0.378 | 0.706 |
| Non-faith folk activities <---Communicative elements | 0.173 | 0.055 | 3.133 | 0.002 | 0.062 | 0.062 | 0.994 | 0.320 | 1.339 | 0.181 |
| Non-folk activities <--- Communicative elements | 0.177 | 0.053 | 3.341 | *** | 0.074 | 0.05 | 1.483 | 0.138 | 1.414 | 0.158 |
| Non-folk activities <--- Activity-oriented elements | 0.11 | 0.058 | 1.884 | 0.06 | 0.054 | 0.061 | 0.882 | 0.378 | 0.665 | 0.506 |
| Non-folk activities <--- Landscape-oriented elements | 0.269 | 0.066 | 4.061 | *** | 0.301 | 0.067 | 4.508 | *** | -0.340 | 0.734 |
| Non-folk activities <--- Accessibility elements | 0.131 | 0.06 | 2.173 | 0.03 | 0.106 | 0.063 | 1.679 | 0.093 | 0.287 | 0.774 |
| Faith-based folk activities <--- Faith-oriented elements | 0.058 | 0.049 | 1.174 | 0.24 | 0.143 | 0.057 | 2.516 | 0.012 | -1.131 | 0.258 |
| Faith-based folk activities <---Communicative elements | 0.010 | 0.054 | 0.178 | 0.859 | -0.052 | 0.056 | -0.925 | 0.355 | 0.797 | 0.425 |
| Faith-based folk activities <---Accessibility elements | 0.064 | 0.063 | 1.022 | 0.307 | 0.079 | 0.071 | 1.114 | 0.265 | -0.158 | 0.874 |
| Faith-based folk activities <---Landscape-oriented elements | 0.005 | 0.067 | 0.074 | 0.941 | 0.115 | 0.072 | 1.582 | 0.114 | -1.118 | 0.263 |
| Social connectedness <--- Activity-oriented elements | 0.068 | 0.049 | 1.381 | 0.167 | 0.109 | 0.05 | 2.195 | 0.028 | -0.586 | 0.558 |
| Social connectedness <--- Landscape-oriented elements | 0.211 | 0.055 | 3.836 | *** | 0.081 | 0.055 | 1.48 | 0.139 | 1.671 | 0.095 |
| Social connectedness <--- Accessibility elements | 0.099 | 0.05 | 1.988 | 0.047 | 0.134 | 0.043 | 3.155 | 0.002 | -0.531 | 0.596 |
| Social connectedness <---Communicative elements | 0.13 | 0.044 | 2.943 | 0.003 | 0.079 | 0.033 | 2.387 | 0.017 | 0.927 | 0.354 |
| Social connectedness <--- Faith-oriented elements | 0.081 | 0.038 | 2.126 | 0.034 | 0.04 | 0.036 | 1.097 | 0.273 | 0.783 | 0.433 |
| Social connectedness <--- Faith-based folk activities | 0.195 | 0.05 | 3.917 | *** | 0.079 | 0.033 | 2.387 | 0.017 | 1.936 | 0.053 |
| Social connectedness <--- Non-faith folk activities | 0.093 | 0.044 | 2.119 | 0.034 | 0.143 | 0.044 | 3.267 | 0.001 | -0.804 | 0.422 |
| Social connectedness <--- Non-folk activities | 0.148 | 0.055 | 2.695 | 0.007 | 0.199 | 0.065 | 3.091 | 0.002 | -0.599 | 0.549 |

*Note. *** p < 0.001; ** p < 0.01; * p < 0.05*
